# Analysis of human lung mast cells by single cell RNA sequencing

**DOI:** 10.3389/fimmu.2023.1151754

**Published:** 2023-03-30

**Authors:** Elin Rönnberg, Avinash Ravindran, Luca Mazzurana, Yitao Gong, Jesper Säfholm, Julie Lorent, Olga Dethlefsen, Ann-Charlotte Orre, Mamdoh Al-Ameri, Mikael Adner, Sven-Erik Dahlén, Joakim S. Dahlin, Jenny Mjösberg, Gunnar Nilsson

**Affiliations:** ^1^ Division of Immunology and Allergy, Department of Medicine Solna, Karolinska Institutet, and Karolinska University Hospital, Stockholm, Sweden; ^2^ Center for Infectious Medicine, Department of Medicine Huddinge, Karolinska Institutet, Stockholm, Sweden; ^3^ Unit for Experimental Asthma and Allergy Research Centre for Allergy Research, The Institute of Environmental Medicine, Karolinska Institutet, Stockholm, Sweden; ^4^ National Bioinformatics Infrastructure Sweden, Science for Life Laboratory, Stockholm University, Stockholm, Sweden; ^5^ Thoracic Surgery, Department of Molecular Medicine and Surgery, Karolinska Institutet, Karolinska University Hospital, Stockholm, Sweden; ^6^ Department of Medical Sciences, Uppsala University, Uppsala, Sweden

**Keywords:** mast cells (MC), single cell RNA sequencing, lung, heterogeniety, chymase (CMA1), cathepsin G (CTSG), carboxypeptidase A3 (CPA3)

## Abstract

Mast cells are tissue-resident cells playing major roles in homeostasis and disease conditions. Lung mast cells are particularly important in airway inflammatory diseases such as asthma. Human mast cells are classically divided into the subsets MC_T_ and MC_TC_, where MC_T_ express the mast cell protease tryptase and MC_TC_ in addition express chymase, carboxypeptidase A3 (CPA3) and cathepsin G. Apart from the disctintion of the MC_T_ and MC_TC_ subsets, little is known about the heterogeniety of human lung mast cells and a deep analysis of their heterogeniety has previously not been performed. We therefore performed single cell RNA sequencing on sorted human lung mast cells using SmartSeq2. The mast cells showed high expression of classical mast cell markers. The expression of several individual genes varied considerably among the cells, however, no subpopulations were detected by unbiased clustering. Variable genes included the protease-encoding transcripts *CMA1* (chymase) and *CTSG* (cathepsin G). Human lung mast cells are predominantly of the MC_T_ subset and consistent with this, the expression of *CMA1* was only detectable in a small proportion of the cells, and correlated moderately to *CTSG*. However, in contrast to established data for the protein, *CPA3* mRNA was high in all cells and the correlation of *CPA3* to *CMA1* was weak.

## Introduction

Mast cells play important roles in the lung both in homeostasis and in disease and they are particularly recognized for their role in asthma (1). Mast cells are found in all different compartments of the human lung; *i.e*., in the epithelium, in smooth muscle bundles, around pulmonary vessels, and in the parenchyma (2). Upon activation, mast cells release their preformed granule content, including histamine, proteases, proteoglycans and some cytokines/chemokine. In addition mast cells also start *de novo* synthesis of lipid mediators and cytokines/chemokines (3). Together, these mediators have multiple effects on the lung causing smooth muscle constriction, mucus production, and edema (1).

Human mast cells are classically divided into the MC_T_ and MC_TC_ subsets depending on the expression of specific proteases. The MC_T_ subset expresses tryptase and MC_TC_ in addition expresses chymase, CPA3 and cathepsin G (4). This distinction has been made with immunohistochemical methods and little is known about the mRNA expression of the proteases at the single cell level. Moreover, the heterogeneity of human lung mast cells appears to be much more diverse, beyond protease expression, including the expression of receptors, enzymes and other markers in different compartments of the lung (5). Recently, using flow cytometry, we demonstrated that human lung mast cell heterogeneity has a continuous nature, rather than distinct populations, both in regard to various cell surface markers but also the classical heterogeneity markers chymase and CPA3 (6). Similarly, a recent scRNAseq study of human mast cells from nasal polyps in patients with chronic rhinosinusitis with nasal polyososis (CRSwNP) also found the heterogeneity of the mast cells to be of a continuous transient nature from MC_T_ to MC_TC_, but the study also found a separate cluster of proliferative mast cells (7).

Several scRNAseq studies using droplet based techniques have been performed on lung tissue in different human diseases, including asthma (8), COPD (9, 10), COVID-19 (11) and lung cancer (12, 13), and a mast cell cluster has been detected in these studies. The mast cell cluster was shown to be enriched in asthma (8) and in one COPD study (9), but the same was not seen in a separate COPD study (10). However, these studies have not been focused on mast cells, and the heterogeneity of the mast cells have therefore not been examined.

In this study, we set out to investigate the heterogeneity of sorted human lung mast cells by scRNAseq using SmartSeq2, a method that provides deeper sequencing than the previous studies that have used droplet-based techniques.

## Materials and methods

### Ethical approval

The local ethics committee approved the collection of lung tissue from patients undergoing lobectomy, and all patients provided informed consent (Regionala Etikprövningsnämnden Stockholm, 2010/181-31/2).

### Cell preparation

Lung tissue was obtained from patients undergoing lobectomy due to lung cancer (age 57–76 year old, three females, one male). All patients were ex smokers and none of them had preoperative chemo- or radiotherapy. Macroscopically healthy tissue distal to the tumour was used. Single-cell suspensions were obtained by digesting the tissue as previously described (14). Briefly, human lung tissue was cut into small pieces and enzymatically digested for 45 min with DNase I and collagenase. Thereafter, the tissue was mechanically disrupted by shearing with a syringe, debris was removed by 30% Percoll centrifugation and red blood cells lysed with an ACK buffer.

### Flow cytometry analysis and cell sorting

For single cell sorting of mast cells the following antibodies were used: V500-CD45 (BD Biosciences, clone HI30), FITC- CD3 (Biolegend, clone SK7), FITC- CD19 (BD Biosciences, clone 4G7), FITC- CD14 (Dako, clone TUK4), FITC- CD1a (Biolegend, clone HI149), FITC- CD123 (Biolegend, clone 6H6), FITC- BDCA2 (Miltenyi Biotech, clone AC144), FITC- TCR α/β (Biolegend, clone IP26), FITC- TCR γ/δ (Biolegend, clone B1), FITC- CD94 (Biolegend, clone DX22), FITC- CD34 (Biolegend, clone 581), PECy5.5-CD117 (Beckman Coulter, clone 104D2D1), and Invitrogen Live/Dead™ Fixable Green Dead Cell Stain kit. FlowJo software was used for flow cytometry data analysis. The mast cells were gated as Live cells, Lineage negative (CD3, CD14, CD19, CD1a, CD123, BDCA2, TCRα/β, TCR γ/δ, CD94, CD34), CD45 positive and CD117 high.

### Single cell RNA sequencing

The isolated human lung mast cells were single cell sorted into 384 well plate containing lysis buffer using a BD FACS Fusion. In total, 332 single mast cells were sorted from 4 lung tissues (83 mast cells per tissue).The quality of cDNA was assessed by agilent high sensitivity DNA assay as previously described (15). Single cell RNA sequencing (scRNA seq) libraries were prepared and sequenced using Smart-seq2 protocol (16) by the SciLifeLab Eukaryotic Single-cell Genomics Facility, Stockholm.

### Data processing and analyses

Data processing and analyses was performed by the National Bioinformatics Infrastructure Sweden (NBIS) and the computations were performed on resources provided by SNIC through Uppsala Multidisciplinary Center for Advanced Computational Science (UPPMAX). Reads were aligned to the reference genome GRCh38 (primary assembly) using star/2.5.3a with default settings (17). Counts per gene (an arbitrary unit of the number or reads that were detected per gene) were calculated for each transcript using featureCounts from subread/1.5.2 using parameters -t exon -g gene_id (18).

The following analysis were performed using R version 3.6.3 (R Core Team 2020). Poor quality cells were excluded. Cells were considered of poor quality if they were outliers (defined using median absolute deviations (MADs)) in at least one of 6 criteria listed (1): cells with percentage of uniquely mapped reads with more than 3 MADs below the median (global median from all plates) (2) cells with percentage of uniquely mapped reads mapping to protein coding regions with more than 3 MADs below the median (global median from all plates) (3) cells with proportions of reads mapped to spike-in transcripts that are more than 3 MADs above the median (global median from all plates) (4) cells with proportions of mitochondrial reads that are more than 3 MADs above the median (global median from all plates) (5) cells with log-library sizes that are more than 3 MADs below the median log-library size of each plate (6) cells which log-transformed number of expressed genes that are more than 3 MADs below the median of each plate.

Genes with no expression (0 count across all mast cells) were removed as well as mitochondrial genes and rRNA genes. Counts were normalized using the logNormCounts function of the scater R/Bioconductor package version 1.14.6 where counts are divided by a cell-specific size factor (19).

Most analyzes were done separately on each plate so no batch correction was performed. The distribution of gene expression of selected marker genes (selected proteases, cell surface receptors and lipid metabolism genes) and the top 20 expressed genes was visualized using violin plots. For each plate, per-gene variance was modeled using spike-ins, highly variable genes across cells were selected using the scran Bioconductor package version 1.14.6 (20). Several dimensionality reduction methods were computed on highly variables genes on the single cell data for each plate separately. Principal Component Analysis (PCA) on the centered normalized log counts (the first 3 principal components were plotted). Uniform Manifold Approximation and Projection (UMAP) on the normalized log counts. t-Distributed Stochastic Neighbor Embedding (tSNE) on the normalized log counts with a perplexity parameter of 100.

The 2-by-2 correlations between CMA1, CPA3 and CTSG was assessed using scatter plots and Spearman’s rank correlation coefficients.

## Results

### scRNA sequencing of human lung mast cells

**Figure 1:**
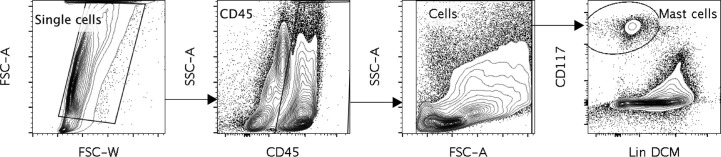
A representative figure of the gating of the human lung mast cells. Cells were gated as Single cells, CD45^+,^ cells selected in the FSC, SSC pattern, CD117 ^high^, dead cell marker (DCM) ^Low^ and Lineage (Lin) ^Low^. The Lineage markers consisted of CD3, CD14, CD19, CD1a, CD123, BDCA2, TCRα/β, TCR γ/δ, CD94 and CD34.

**Figure 2:**
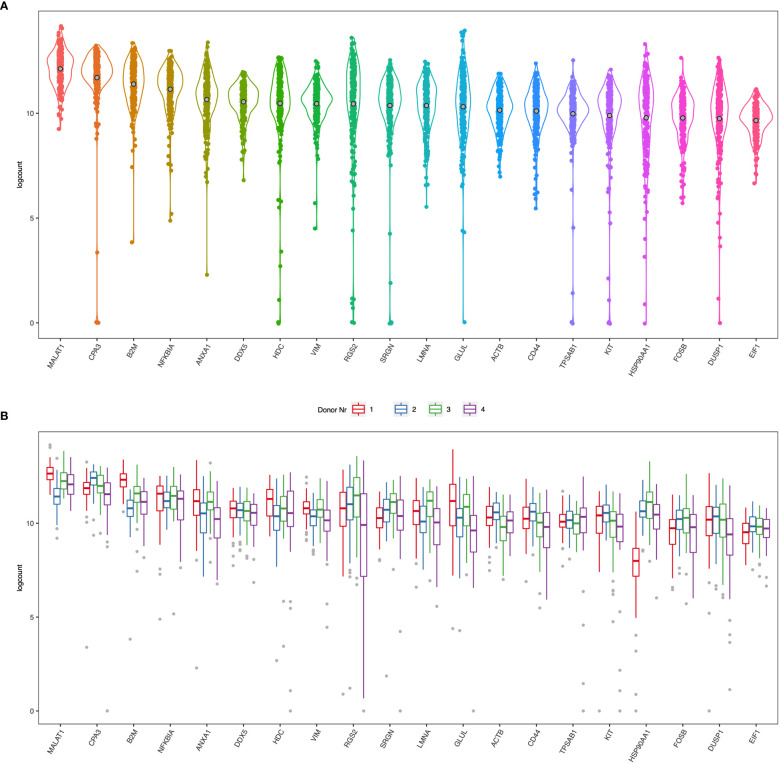
**(A)** Violin plots of the top 20 ranked genes in the dataset from all four donors **(B)** Boxplots of the top 20 ranked genes separated by each donor.

Human lung mast cell heterogeneity has been thoroughly examined using immunohistochemistry and flow cytometry (5, 6). However, an unbiased high throughput investigation into human lung mast cell heterogeneity has not been performed. We therefore set out to perform scRNAseq on sorted human lung mast cells. The mast cells were gated as Live Lin^-^ CD45^+^ CD117^high^ (**Figure 1**) and scRNAseq was performed using the Smart-seq2 protocol. Among the genes with the highest counts (**Figures 2A, B**, **Supplementary Figure S1**) were many known mast cell genes such as the receptor for stem cell factor *KIT*, that is needed for mast cell maturation and survival, the mast cell proteases tryptase (*TPSAB1*) and carboxypeptidase A3 (*CPA3*), the proteoglycan serglycin (*SRGN*) that is needed for the packing of biogenic amines (histamine, serotonin, dopamine) and proteases in the secretory granules (21–23), and histidine decarboxylase (*HDC*) that is the rate limiting enzyme in the biosynthesis of histamine. However, we also noted four cells that had low to undetectable level of several mast cell signature genes, including TPSAB1, TPSB2, CPA3, HDC and IL1RL1, indicating that these could be contaminating non-mast cells (data not shown).

Many of the highest expressed genes were also involved in basic functions of cells and therefore highly expressed in most cells such as β2 microglobulin (*B2M*), β-actin (*ACTB*), vimentin (*VIM*), lamin (*LMNA*), glutamine synthase (*GLUL*). *MALAT1* is a long non-coding RNA that is frequently high in poly-A captured RNA sequencing data (24, 25). There are also several genes involved in transcription and translation, such as transcription factors *NFKBIA* and *FOSB*, regulator of transcription and splicing *DDX5*, and regulator of translation initiation *EIF1*, regulator of the transcription complex and chaperone *HSP90AA1.* Annexin A1 (*ANX1*) is a protein that binds phospholipids and inhibits phospholipase A2. Synthesis and secretion of annexin A1 is enhanced by glucocorticoids (26, 27) and mast cell activation is inhibited by annexin A1 (28). Another highly expressed gene was CD44, a ubiquitously expressed adhesion molecule with several ligands, including hyaluronic acid, that previously has been shown to be expressed by human mast cells (29, 30).

### Heterogenous expression of mast cell genes

**Figure 3:**
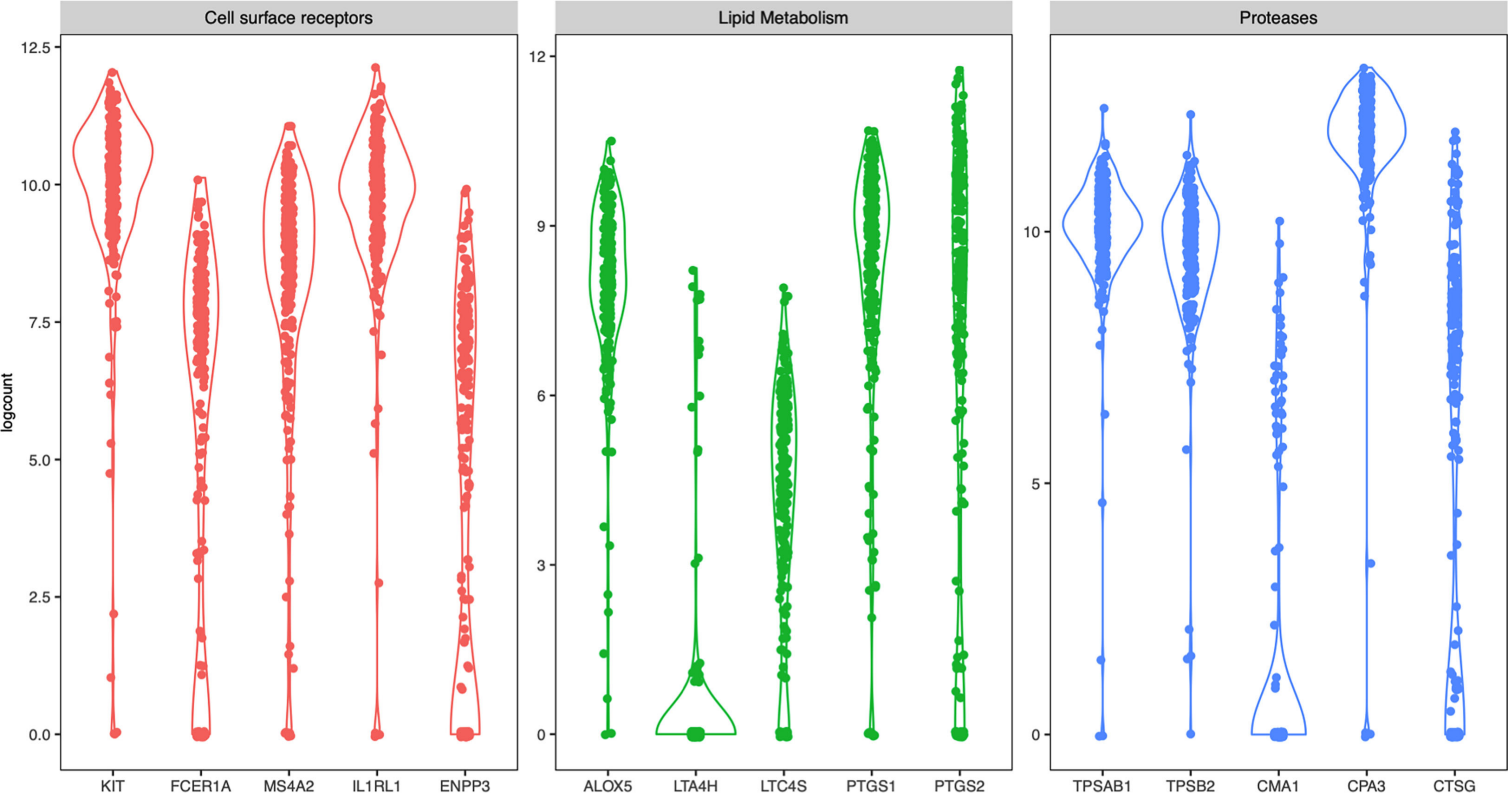
Violin plots of selected mast cells signature genes from all four donors.

Next, we specifically examined the heterogeneity of expression of selected mast-cell related genes. The cell surface receptors *KIT* and the IL-33 receptor ST2 (*IL1RL1*) were as expected high in most cells, while the two of the subunits of the FcεRI receptor (*FCER1A* and *MS4A2*) and CD203c (*ENPP3*) had a more diverse expression pattern (**Figure 3**). Of the enzymes involved in lipid metabolism, arachidonate 5-lipoxygenase (*ALOX5*, also known as 5-LOX) was highly detectable in most cells (**Figure 3**). 5-LOX converts arachidonic acid to leukotriene A4 (LTA4), that is further metabolized to LTB_4_ by LTA4 hydrolase (LTA4H) or to LTC_4_ by leukotriene C4 synthase (LTC4S). In the human lung mast cells *LTA4H* was only detectable in a small proportion of the cells, while *LTC4S* was detectable in a higher proportion of the cells and had a variable expression pattern (**Figure 3**). Both cyclooxygenases that converts arachidonic acid to prostaglandin endoperoxide H2 (a precursor for the prostaglandins D_2_, E_2_ and F_2_), prostaglandin-endoperoxide synthase 1 (*PTGS1*- also known as cyclooxygenase 1 (COX-1)) and *PTGS2* (also known as COX-2) were variably expressed (**Figure 3**). Of the proteases, the tryptases (*TPSAB1* and *TPSB2*) and CPA3 were high in most cells whereas chymase (*CMA1*) and Cathepsin G (*CTSG*) had a variable expression pattern (**Figure 3**).

### No distinct mast cell subsets were detected

**Figure 4:**
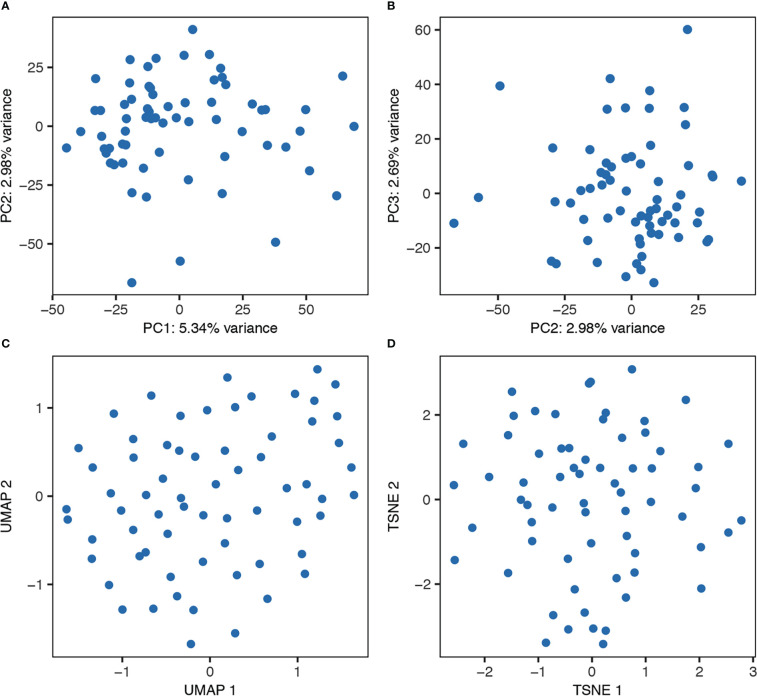
Unbiased clustering of the cells from one representative donor using PCA **(A, B)**, UMAP **(C)**, and tSNE **(D)**.

**Figure 5:**
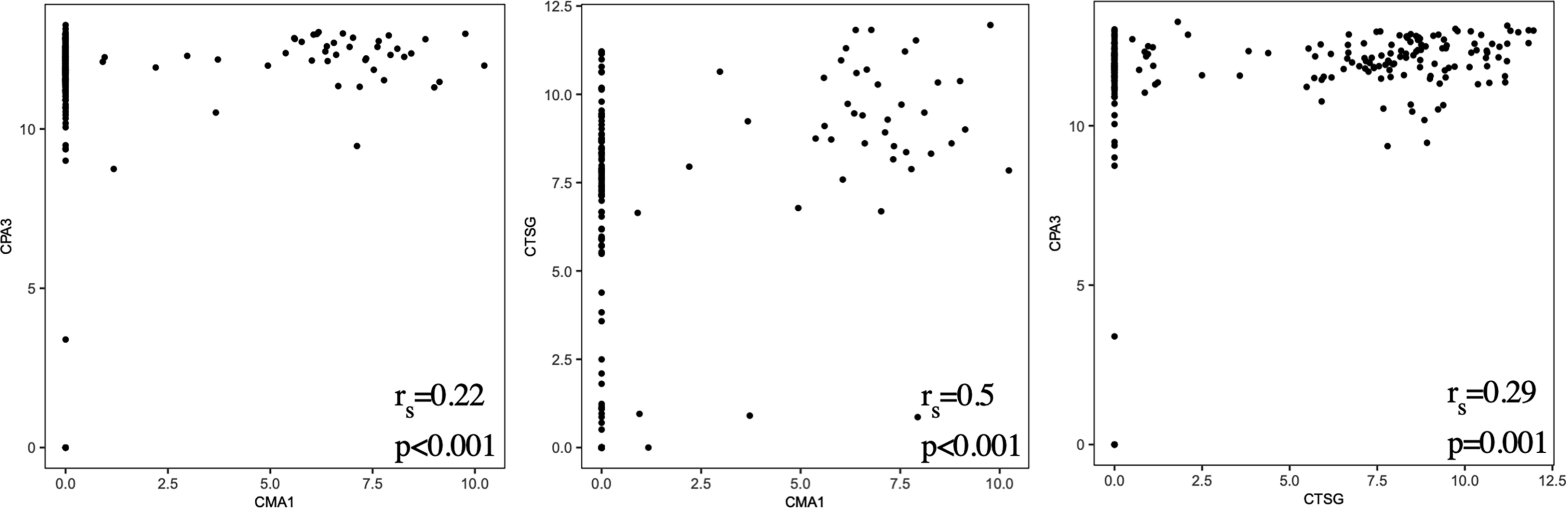
Scatter plots of *CPA3/CMA1/CTSG* expression from all four donors and Spearman’s rank correlation coefficients (r_s_).

To examine possible subsets of human lung mast cells we used three different methods to cluster the cells based on the scRNAseq data: PCA, UMAP and tSNE plots. None of these three methods provided any obvious sub-clusters (one representative donor in **Figure 4** and the additional three donor in **Supplementary Figure S2**). We noted that there were a few cells that positioned apart from the main cell cluster in two donors, these cells were mainly the suspected non- mast cells that had low to undectectable levels of the mast cell signature genes (**Supplementary Figures S2A, C**). To specifically examine the classical mast cell subsets MC_T_ and MC_TC,_ we examined the correlation in expression between the proteases that are present in the MC_TC_ subset, that is chymase, CPA3 and cathepsin G. To the contrary of the protein (6), the *CPA3* mRNA was highly detectable in all cells, and the correlation to *CMA1* and *CTSG* was weak (**Figure 5**). In contrast, the chymase, *CMA1* expression was only detected in a small fraction of the cells that also had relatively high *CTSG* expression, but on the other hand there where cells with high *CTSG* that did not have detectable *CMA1* expression. Thus, *CMA1* correlated moderately with that of *CTSG* with a Spearman’s rank correlation coefficient of 0.5 (**Figure 5**). *CTSG* and *CMA1* did not show correlation (moderate or high, r_s_>0.4) to any other genes in the dataset (**Supplementary Tables S1, S2**).

## Discussion

We have investigated the heterogeneity of human lung mast cells using SmartSeq2, a method that provides deep sequencing of single cells. Among the genes with the highest counts were several mast cell genes, including *KIT*, tryptase (*TPSAB1*), carboxypeptidase A3 (*CPA3*), serglycin (*SRGN*), histidine decarboxylase (*HDC*), verifying the identity of the cells as mast cells.

The expression of two subunits of the FcεRI receptor was shown to be variable (**Figure 3**), they did not however correlate to each other (data not shown). Variable surface expression of FcεRI has previously been reported on human lung mast cells as well as for human skin mast cells (6, 31). The expression of the FcεRI protein on mast cells has also been shown to vary between different lung compartments, i.e. central airways, small airways, parenchyma, and pulmonary vessels, with particularly low expression on the parenchymal mast cells (5). The low parenchymal expression of FcεRI in healthy subjects is, however, increased in patients with atopic uncontrolled asthma (32). The underlying mechanism for the varying FcεRI expression is not completely known. The FcεRI mRNA expression has been shown to be regulated by IL-4 (33) and IL-33 (34, 35), and the receptor is stabilized on the cell-surface by the presence of IgE (36), but other mechanisms might also be involved in the regulation.

The main prostanoid released from human mast cells upon activation is PGD_2_ (37–39), which have potent biological effects in the lung, such as broncoconstriction (40), vasodilation (41), and activation and recruitment of group 2 innate lymphoid cells (42). The first step in the biosynthesis of PGD_2,_ is catalyzed by two enzymes COX-1 (*PTGS1*) and COX-2 (*PTGS2*). In our study both genes encoding for COX-1 and -2, *PTGS1* and *PTGS2*, were variably expressed (**Figure 3**). However, the expression of the two genes did not correlate to each other (data not shown). Additionally, in nasal polyps, *PTGS2* is enriched in the MC_TC_ cluster (7), which we could not observe in HLMC. In human mast cells, PGD_2_ biosynthesis have been shown to be entirely dependent on COX-1 (37, 43), so, why *PTGS2* (COX-2) is transcribed in these cells and under what conditions it is enzymatically active remains to be elucidated.

Human mast cells have been shown to be heterogeneous when it comes to the presence of proteases and thus divided into MC_T_ and MC_TC_ depending on their expression of chymase (*CMA1*). The MC_TC_ subset has in addition also been shown to contain CPA3 and cathepsin G (44, 45). Human lung mast cells are predominantly of the MC_T_ subtype, and consistent with this *CMA1* was only detectable in a small proportion of the cells (**Figures 3**, **5**). We found high expression of *CPA3* mRNA in all lung mast cells and the correlation of this expression to *CMA1* was weak (**Figures 3**, **5**). In contrast, CPA3 and chymase proteins strongly correlate in human lung mast cells as judged by flow cytometry (6). This discrepancy of CPA3 mRNA and protein has also been shown in human lung mast cells using RNAscope and immunohistochemistry (46). When the gene for the equivalent to chymase in mice (based on sequence alignment), mouse mast cells protease- 5 (mMCP-5), is deleted the granule storage of CPA3 is lost, despite the *CPA3* mRNA expression being unaffected (47). Similarly, if the *CPA3* gene is deleted, the granule storage of the mMCP-5 protein is lost (48). However, when CPA3 is mutated to render it inactive without deletion of the CPA3 protein, the storage of mMCP-5 is unaffective (49). Thus, in mice mMCP-5 and CPA3 protein are interdependent on each other for their granule storage in mast cells. One can speculate that the same situation could be true for human CPA3, that chymase needs to be present for the CPA3 protease to be stored in the granules. Supporting this hypothesis is the finding that when comparing lung and skin mast cells, skin mast cells have higher expression of chymase and store more CPA3 protein in spite of expressing lower level of *CPA3* mRNA. This theory does however raise the question; why do the cells express high levels of the mRNA if the protein is not stored? Is there a high CPA3 turnover or perhaps a continuous release of CPA3 in the MC_T_ subtype? Further studies are needed to answer these questions.

The mast cells in this study comes from lobectomies performed on cancer patients. The tissue used was macroscopically healthy distal to the tumour, nevertheless it is of course possible that the disease has affected the included mast cells. In this context it is worth noting that CPA3 counts were high (**Figure 3**) and CPA3 expression has been shown to correlate to decreased lung function in patients with severe chronic obstructive pulmonary disease (COPD) and idiopathic lung fibrosis (IPF) (50).

The aim of the present study was to investigate human lung mast cell heterogeneity in more depth using SmartSeq2 single-cell RNA sequencing. However, we could not detect any obvious transcriptional different subpopulations of the human lung mast cells (**Figure 4**). There could be several explanations for this. The human lung mast cells could be homogenous in nature but it could also be that the number of mast cells analyzed are too low to distinguish small subsets. The facts that the mRNA of one of the proteases present in the MC_TC_ subtype, *CPA3*, does not correlate to the protein, and that no other genes then *CTSG* show a correlation (r_s_>0.4) to *CMA1*, likely contribute to the inability to distinguish the classical mast cell subsets based on unbiased clustering. Furthermore, our study covered mainly mast cells in the parenchyma and it might be that mast cells in different compartments of the lung show greater heterogeneity. Thus, additional analyses including higher number of single mast cells from different lung compartments will be instrumental to decipher further the heterogeneity of human lung mast cells.

## Data availability statement

The data presented in the study are deposited in the Gene Expression Omnibus (GEO) repository, accession number GSE227712.

## Ethics statement

The studies involving human participants were reviewed and approved by Regionala Etikprövningsnämden i Stockholm. The patients/participants provided their written informed consent to participate in this study.

## Author contributions

GN, JM, AR, ER, and JD, conceived and designed the studies. AR, and LM, performed the experiments. JL, OD, YG, ER, and JD analyzed the data. JS, A-CO, MA-A, MA and S-ED provided samples. ER, AR and GN wrote the manuscript draft. All authors contributed to the article and approved the submitted version.

## References

[1] Bradding P Arthur G. Mast cells in asthma–state of the art. Clin Exp Allergy (2016) 46(2):194–263. doi: 10.1111/cea.12675 26567481

[2] Erjefalt JS. Mast cells in human airways: The culprit? Eur Respir Rev (2014) 23(133):299–307. doi: 10.1183/09059180.00005014 25176966 PMC9487311

[3] Dahlin JS Maurer M Metcalfe DD Pejler G Sagi-Eisenberg R Nilsson G. The ingenious mast cell: Contemporary insights into mast cell behavior and function. Allergy (2021) 77(1):83–99. doi: 10.1111/all.14881 33955017

[4] Irani AA Schechter NM Craig SS DeBlois G Schwartz LB. Two types of human mast cells that have distinct neutral protease compositions. Proc Natl Acad Sci U.S.A. (1986) 83(12):4464–8. doi: 10.1073/pnas.83.12.4464 PMC323754 3520574

[5] Andersson CK Mori M Bjermer L Lofdahl CG Erjefalt JS. Novel site-specific mast cell subpopulations in the human lung. Thorax (2009) 64(4):297–305. doi: 10.1136/thx.2008.101683 19131451

[6] Ronnberg E Boey DZH Ravindran A Safholm J Orre AC Al-Ameri M. Immunoprofiling reveals novel mast cell receptors and the continuous nature of human lung mast cell heterogeneity. Front Immunol (2021) 12:804812. doi: 10.3389/fimmu.2021.804812 35058936 PMC8764255

[7] Dwyer DF Ordovas-Montanes J Allon SJ Buchheit KM Vukovic M Derakhshan T. Human airway mast cells proliferate and acquire distinct inflammation-driven phenotypes during type 2 inflammation. Sci Immunol (2021) 6(56):eabb7221. doi: 10.1126/sciimmunol.abb7221 33637594 PMC8362933

[8] Vieira Braga FA Kar G Berg M Carpaij OA Polanski K Simon LM. A cellular census of human lungs identifies novel cell states in health and in asthma. Nat Med (2019) 25(7):1153–63. doi: 10.1038/s41591-019-0468-5 31209336

[9] Sauler M McDonough JE Adams TS Kothapalli N Barnthaler T Werder RB. Characterization of the copd alveolar niche using single-cell rna sequencing. Nat Commun (2022) 13(1):494. doi: 10.1038/s41467-022-28062-9 35078977 PMC8789871

[10] Li X Noell G Tabib T Gregory AD Trejo Bittar HE Vats R. Single cell rna sequencing identifies Igfbp5 and qki as ciliated epithelial cell genes associated with severe copd. Respir Res (2021) 22(1):100. doi: 10.1186/s12931-021-01675-2 33823868 PMC8022543

[11] Delorey TM Ziegler CGK Heimberg G Normand R Yang YM Segerstolpe A. Covid-19 tissue atlases reveal sars-Cov-2 pathology and cellular targets. Nature (2021) 595(7865):107. doi: 10.1038/s41586-021-03570-8 33915569 PMC8919505

[12] Wu F Fan J He Y Xiong A Yu J Li Y. Single-cell profiling of tumor heterogeneity and the microenvironment in advanced non-small cell lung cancer. Nat Commun (2021) 12(1):2540. doi: 10.1038/s41467-021-22801-0 33953163 PMC8100173

[13] Lu T Yang X Shi Y Zhao M Bi G Liang J. Single-cell transcriptome atlas of lung adenocarcinoma featured with ground glass nodules. Cell Discovery (2020) 6:69. doi: 10.1038/s41421-020-00200-x 33083004 PMC7536439

[14] Ravindran A Ronnberg E Dahlin JS Mazzurana L Safholm J Orre AC. An optimized protocol for the isolation and functional analysis of human lung mast cells. Front Immunol (2018) 9:2193. doi: 10.3389/fimmu.2018.02193 30344519 PMC6183502

[15] Bjorklund AK Forkel M Picelli S Konya V Theorell J Friberg D. The heterogeneity of human Cd127(+) innate lymphoid cells revealed by single-cell rna sequencing. Nat Immunol (2016) 17(4):451–60. doi: 10.1038/ni.3368 26878113

[16] Picelli S Faridani OR Bjorklund AK Winberg G Sagasser S Sandberg R. Full-length rna-seq from single cells using smart-Seq2. Nat Protoc (2014) 9(1):171–81. doi: 10.1038/nprot.2014.006 24385147

[17] Dobin A Davis CA Schlesinger F Drenkow J Zaleski C Jha S. Star: Ultrafast universal rna-seq aligner. Bioinformatics (2013) 29(1):15–21. doi: 10.1093/bioinformatics/bts635 23104886 PMC3530905

[18] Liao Y Smyth GK Shi W. The subread aligner: Fast, accurate and scalable read mapping by seed-and-Vote. Nucleic Acids Res (2013) 41(10):e108. doi: 10.1093/nar/gkt214 23558742 PMC3664803

[19] McCarthy DJ Campbell KR Lun AT Wills QF. Scater: Pre-processing, quality control, normalization and visualization of single-cell rna-seq data in r. Bioinformatics (2017) 33(8):1179–86. doi: 10.1093/bioinformatics/btw777 PMC5408845 28088763

[20] Lun AT McCarthy DJ Marioni JC. A step-by-Step workflow for low-level analysis of single-cell rna-seq data with bioconductor. F1000Res (2016) 5:2122. doi: 10.12688/f1000research.9501.2 27909575 PMC5112579

[21] Ronnberg E Calounova G Pejler G. Mast cells express tyrosine hydroxylase and store dopamine in a serglycin-dependent manner. Biol Chem (2012) 393(1-2):107–12. doi: 10.1515/BC-2011-220 22628305

[22] Ringvall M Ronnberg E Wernersson S Duelli A Henningsson F Abrink M. Serotonin and histamine storage in mast cell secretory granules is dependent on serglycin proteoglycan. J Allergy Clin Immunol (2008) 121(4):1020–6. doi: 10.1016/j.jaci.2007.11.031 18234316

[23] Abrink M Grujic M Pejler G. Serglycin is essential for maturation of mast cell secretory granule. J Biol Chem (2004) 279(39):40897–905. doi: 10.1074/jbc.M405856200 15231821

[24] Aissa AF Islam A Ariss MM Go CC Rader AE Conrardy RD. Single-cell transcriptional changes associated with drug tolerance and response to combination therapies in cancer. Nat Commun (2021) 12(1):1628. doi: 10.1038/s41467-021-21884-z 33712615 PMC7955121

[25] Zhang B Arun G Mao YS Lazar Z Hung G Bhattacharjee G. The lncrna Malat1 is dispensable for mouse development but its transcription plays a cis-regulatory role in the adult. Cell Rep (2012) 2(1):111–23. doi: 10.1016/j.celrep.2012.06.003 PMC3408587 22840402

[26] Hannon R Croxtall JD Getting SJ Roviezzo F Yona S Paul-Clark MJ. Aberrant inflammation and resistance to glucocorticoids in annexin 1-/- mouse. FASEB J (2003) 17(2):253–5. doi: 10.1096/fj.02-0239fje 12475898

[27] Peers SH Smillie F Elderfield AJ Flower RJ. Glucocorticoid-and non-glucocorticoid induction of lipocortins (Annexins) 1 and 2 in rat peritoneal leucocytes in vivo. Br J Pharmacol (1993) 108(1):66–72. doi: 10.1111/j.1476-5381.1993.tb13441.x 8428216 PMC1907693

[28] Sinniah A Yazid S Bena S Oliani SM Perretti M Flower RJ. Endogenous annexin-A1 negatively regulates mast cell-mediated allergic reactions. Front Pharmacol (2019) 10:1313. doi: 10.3389/fphar.2019.01313 31798445 PMC6865276

[29] Valent P Majdic O Maurer D Bodger M Muhm M Bettelheim P. Further characterization of surface membrane structures expressed on human basophils and mast cells. Int Arch Allergy Appl Immunol (1990) 91(2):198–203. doi: 10.1159/000235115 1971264

[30] Girodet PO Ozier A Trian T Begueret H Ousova O Vernejoux JM. Mast cell adhesion to bronchial smooth muscle in asthma specifically depends on Cd51 and Cd44 variant 6. Allergy (2010) 65(8):1004–12. doi: 10.1111/j.1398-9995.2009.02308.x 20121756

[31] Babina M Guhl S Artuc M Trivedi NN Zuberbier T. Phenotypic variability in human skin mast cells. Exp Dermatol (2016) 25(6):434–9. doi: 10.1111/exd.12924 26706922

[32] Andersson CK Bergqvist A Mori M Mauad T Bjermer L Erjefalt JS. Mast cell-associated alveolar inflammation in patients with atopic uncontrolled asthma. J Allergy Clin Immunol (2011) 127(4):905–12 e1-7. doi: 10.1016/j.jaci.2011.01.022 21388666

[33] Toru H Ra C Nonoyama S Suzuki K Yata J Nakahata T. Induction of the high-affinity ige receptor (Fc epsilon ri) on human mast cells by il-4. Int Immunol (1996) 8(9):1367–73. doi: 10.1093/intimm/8.9.1367 8921414

[34] Ronnberg E Ghaib A Ceriol C Enoksson M Arock M Safholm J. Divergent effects of acute and prolonged interleukin 33 exposure on mast cell ige-mediated functions. Front Immunol (2019) 10:1361. doi: 10.3389/fimmu.2019.01361 31275312 PMC6593472

[35] Babina M Wang Z Franke K Guhl S Artuc M Zuberbier T. Yin-yang of il-33 in human skin mast cells: Reduced degranulation, but augmented histamine synthesis through P38 activation. J Invest Dermatol (2019) 139(7):1516–25.e3. doi: 10.1016/j.jid.2019.01.013 30684550

[36] Kraft S Kinet JP. New developments in fcepsilonri regulation, function and inhibition. Nat Rev Immunol (2007) 7(5):365–78. doi: 10.1038/nri2072 17438574

[37] Johnsson AK Choi JH Ronnberg E Fuchs D Kolmert J Hamberg M. Selective inhibition of prostaglandin D2 biosynthesis in human mast cells to overcome need for multiple receptor antagonists: Biochemical consequences. Clin Exp Allergy (2021) 51(4):594–603. doi: 10.1111/cea.13831 33449404

[38] Lewis RA Soter NA Diamond PT Austen KF Oates JA Roberts LJ 2nd. Prostaglandin D2 generation after activation of rat and human mast cells with anti-ige. J Immunol (1982) 129(4):1627–31. doi: 10.4049/jimmunol.129.4.1627 6809826

[39] Peters SP MacGlashan DW Jr. Schulman ES Schleimer RP Hayes EC Rokach J. Arachidonic acid metabolism in purified human lung mast cells. J Immunol (1984) 132(4):1972–9. doi: 10.4049/jimmunol.132.4.1972 6199420

[40] Hardy CC Robinson C Tattersfield AE Holgate ST. The bronchoconstrictor effect of inhaled prostaglandin D2 in normal and asthmatic men. N Engl J Med (1984) 311(4):209–13. doi: 10.1056/NEJM198407263110401 6588293

[41] Alving K Matran R Lundberg JM. The possible role of prostaglandin D2 in the long-lasting airways vasodilatation induced by allergen in the sensitized pig. Acta Physiol Scand (1991) 143(1):93–103. doi: 10.1111/j.1748-1716.1991.tb09204.x 1957709

[42] Xue L Salimi M Panse I Mjosberg JM McKenzie AN Spits H. Prostaglandin D2 activates group 2 innate lymphoid cells through chemoattractant receptor-homologous molecule expressed on Th2 cells. J Allergy Clin Immunol (2014) 133(4):1184–94. doi: 10.1016/j.jaci.2013.10.056 PMC3979107 24388011

[43] Baothman BK Smith J Kay LJ Suvarna SK Peachell PT. Prostaglandin D2 generation from human lung mast cells is catalysed exclusively by cyclooxygenase-1. Eur J Pharmacol (2018) 819:225–32. doi: 10.1016/j.ejphar.2017.12.005 29225187

[44] Schechter NM Irani AM Sprows JL Abernethy J Wintroub B Schwartz LB. Identification of a cathepsin G-like proteinase in the mctc type of human mast cell. J Immunol (1990) 145(8):2652–61. doi: 10.4049/jimmunol.145.8.2652 2212656

[45] Irani AM Goldstein SM Wintroub BU Bradford T Schwartz LB. Human mast cell carboxypeptidase. Selective Localization to Mctc Cells J Immunol (1991) 147(1):247–53. 2051021

[46] Siddhuraj P Clausson CM Sanden C Alyamani M Kadivar M Marsal J. Lung mast cells have a high constitutive expression of carboxypeptidase A3 mrna that is independent from granule-stored Cpa3. Cells (2021) 10(2):309. doi: 10.3390/cells10020309 33546258 PMC7913381

[47] Stevens RL McNeil HP Wensing LA Shin K Wong GW Hansbro PM. Experimental arthritis is dependent on mouse mast cell protease-5. J Biol Chem (2017) 292(13):5392–404. doi: 10.1074/jbc.M116.773416 PMC5392683 28193842

[48] Feyerabend TB Hausser H Tietz A Blum C Hellman L Straus AH. Loss of histochemical identity in mast cells lacking carboxypeptidase a. Mol Cell Biol (2005) 25(14):6199–210. doi: 10.1128/MCB.25.14.6199-6210.2005 PMC1168831 15988029

[49] Schneider LA Schlenner SM Feyerabend TB Wunderlin M Rodewald HR. Molecular mechanism of mast cell mediated innate defense against endothelin and snake venom sarafotoxin. J Exp Med (2007) 204(11):2629–39. doi: 10.1084/jem.20071262 PMC2118486 17923505

[50] Siddhuraj P Jonsson J Alyamani M Prabhala P Magnusson M Lindstedt S. Dynamically upregulated mast cell Cpa3 patterns in chronic obstructive pulmonary disease and idiopathic pulmonary fibrosis. Front Immunol (2022) 13:924244. doi: 10.3389/fimmu.2022.924244 35983043 PMC9378779

