# Supplementary material for: Analysis of human lung mast cells by single cell RNA sequencing
Source: Front Immunol. 2023 Mar 30;14:1151754. doi: 10.3389/fimmu.2023.1151754 (PMC10100501; doi:10.3389/fimmu.2023.1151754)
Supplement: Supplementary file 1 [file DataSheet_1.pdf]

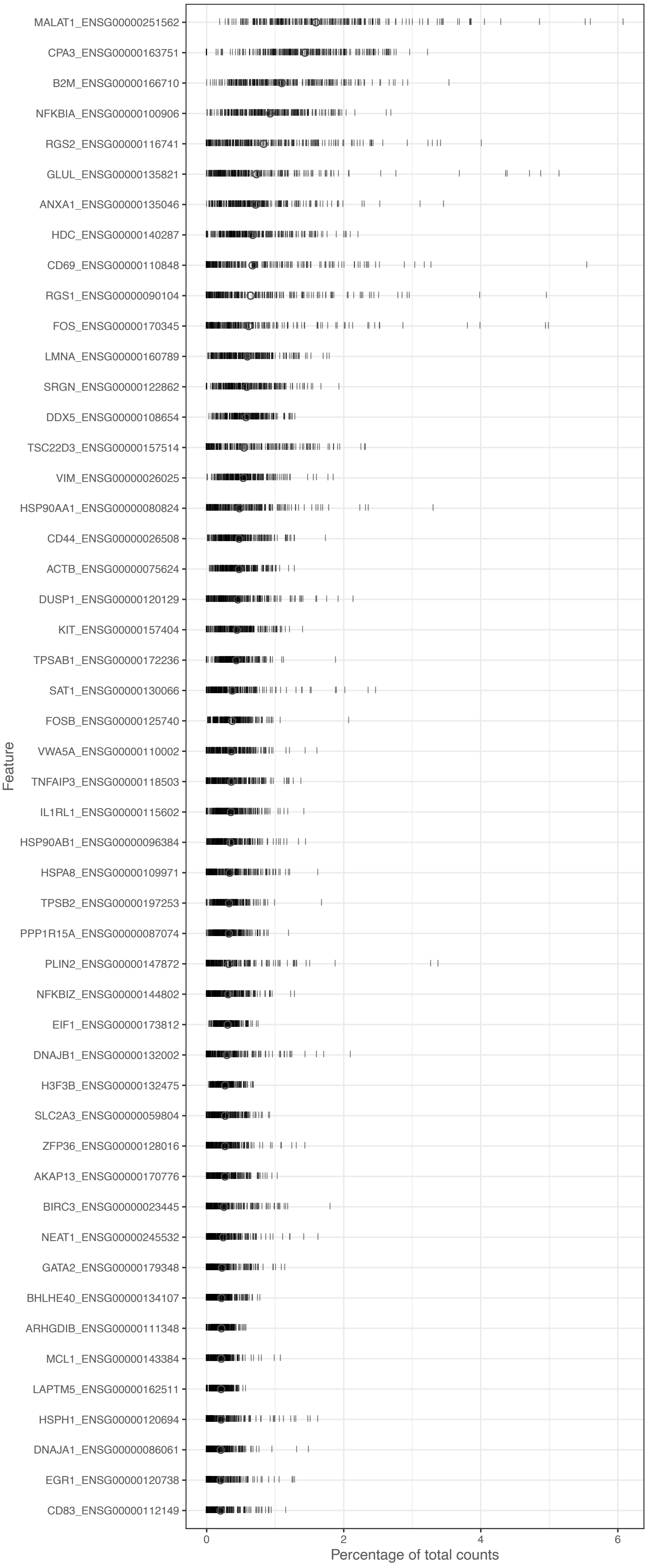

Supplementary Figure S1. Top 50 ranked genes in the dataset

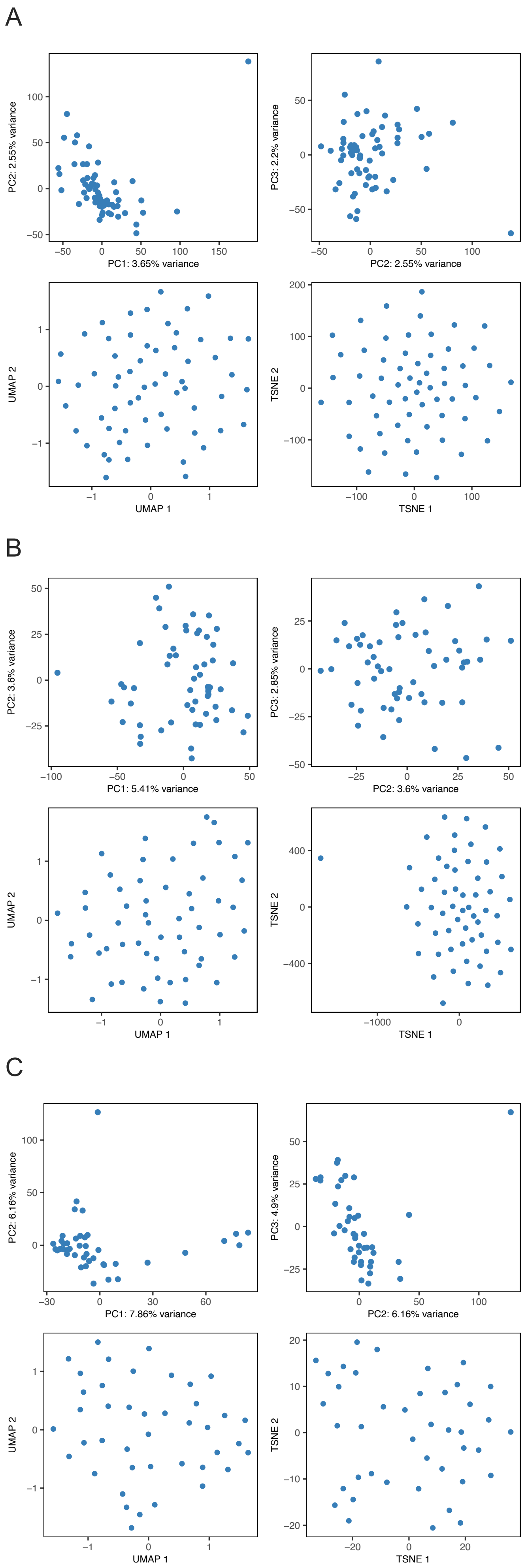

Supplementry Figure S2. Unbiased clustering of the cells from three separate donors (A, B and C) using PCA, UMAP , and tSNE.
