# Supplementary material for: Analysis of human lung mast cells by single cell RNA sequencing
Source: Front Immunol. 2023 Mar 30;14:1151754. doi: 10.3389/fimmu.2023.1151754 (PMC10100501; doi:10.3389/fimmu.2023.1151754)
Supplement: Supplementary file 2 [file Table_1.docx]

Supplementary Table S1. Spearman correlations of *CTSG* (with r_s_>0.25 or r_s_<-0.25 and P<0.05) to other genes in the scRNAseq dataset

|  | **r_s_ value** | P value |  | **r_s_ value** | P value |
| --- | --- | --- | --- | --- | --- |
| **CMA1** | 0.50 | <0.001 | **AP000902.1** | -0.36 | <0.001 |
| **ATP8B4** | 0.38 | <0.001 | **RPS4Y1** | -0.35 | <0.001 |
| **TSC22D3** | 0.32 | 0.014 | **AHNAK2** | -0.31 | <0.001 |
| **HSP90AA1** | 0.32 | 0.006 | **DDX3Y** | -0.29 | <0.001 |
| **MIR3681HG** | 0.30 | <0.001 | **RPS14** | -0.28 | <0.001 |
| **AL662860.1** | 0.29 | 0.001 | **ANKRD20A11P** | -0.28 | <0.001 |
| **ADCYAP1** | 0.29 | <0.001 | **CD69** | -0.28 | <0.001 |
| **CPA3** | 0.29 | 0.001 | **USP9Y** | -0.27 | <0.001 |
| **AP000894.2** | 0.28 | 0.003 | **AC005261.1** | -0.26 | <0.001 |
| **HSP90AA6P** | 0.28 | 0.005 | **AC005884.2** | -0.26 | <0.001 |
| **IGKJ3** | 0.27 | 0.003 | **SCIN** | -0.25 | <0.001 |
| **KIF13B** | 0.27 | 0.001 | **SLC25A4** | -0.25 | <0.001 |
| **DNAJA1** | 0.26 | 0.014 | **NMI** | -0.25 | <0.001 |
| **HSPH1** | 0.26 | 0.008 |  |  |  |
| **IL18R1** | 0.26 | 0.017 |  |  |  |
| **HSPD1P1** | 0.26 | 0.008 |  |  |  |
| **AC120498.6** | 0.25 | 0.001 |  |  |  |
| **P4HB** | 0.25 | 0.008 |  |  |  |

Supplementary Table S2. Spearman correlations of *CMA1* (with r_s_>0.25 or r_s_<-0.25 and P<0.05) to other genes in the scRNAseq dataset.

|  | **r_s_ value** | **P value** |  | **r_s_ value** | **P value** |
| --- | --- | --- | --- | --- | --- |
| **CTSG** | 0.50 | <0.001 | **CNIH1** | -0.25 | <0.001 |
| **MIR3681HG** | 0.34 | <0.001 |  |  |  |
| **AC007879.4** | 0.27 | <0.001 |  |  |  |
| **BCAR1** | 0.27 | <0.001 |  |  |  |
| **TLE1P1** | 0.27 | <0.001 |  |  |  |
| **APOBEC3D** | 0.27 | <0.001 |  |  |  |
| **CRYGS** | 0.26 | <0.001 |  |  |  |
| **ATP8B4** | 0.26 | <0.001 |  |  |  |
| **AP000787.1** | 0.26 | <0.001 |  |  |  |
| **MIR6729** | 0.26 | <0.001 |  |  |  |
| **BTBD10P1** | 0.26 | <0.001 |  |  |  |
| **SSC5D** | 0.25 | <0.001 |  |  |  |
| **SMARCAD1** | 0.25 | <0.001 |  |  |  |
| **AC009093.10** | 0.25 | <0.001 |  |  |  |
| **ANKRD18DP** | 0.25 | <0.001 |  |  |  |
| **AC015883.1** | 0.25 | <0.001 |  |  |  |
| **AL136038.5** | 0.25 | <0.001 |  |  |  |
| **AC004917.1** | 0.25 | <0.001 |  |  |  |
| **AL132800.1** | 0.25 | <0.001 |  |  |  |
| **AC007879.3** | 0.25 | <0.001 |  |  |  |
| **DRD2** | 0.25 | <0.001 |  |  |  |
